# Zoledronic acid affects the process of *Porphyromonas gingivalis* infecting oral mucosal epithelial barrier: An *in-vivo* and *in-vitro* study

**DOI:** 10.3389/fcimb.2023.1104826

**Published:** 2023-03-28

**Authors:** Hanyu Sun, Pugeng Li, Qingci Kong, Feilong Deng, Xiaolin Yu

**Affiliations:** ^1^ Hospital of Stomatology, Guanghua School of Stomatology, Sun Yat-Sen University, Guangzhou, China; ^2^ Guangdong Provincial Key Laboratory of Stomatology, Sun Yat-Sen University, Guangzhou, China

**Keywords:** *Porphyromonas gingivalis*, zoledronic acid, bacterial infection, gingival epithelial cells, pro-inflammatory cytokines

## Abstract

Zoledronic acid (ZA), one of the commonly used bisphosphonates, is mainly used for bone-metabolic diseases. Studies proved that ZA has adverse effects on oral soft tissues. As the first line of innate immunity, the gingival epithelium could be infected by periodontal pathogens, which is a key process of the initiation of periodontal diseases. Yet, how ZA affects the periodontal pathogens infecting the epithelial barrier remains unclear. This study aimed to investigate the influences of ZA on the process of *Porphyromonas gingivalis* (*P. gingivalis*) infecting the gingival epithelial barrier *via in-vitro* and *in-vivo* experiments. In the *in-vitro* experiments, under the condition of different concentrations of ZA (0, 1, 10, and 100 μM), *P. gingivalis* was used to infect human gingival epithelial cells (HGECs). The infections were detected by transmission electron microscope and confocal laser scanning microscope. Besides, the internalization assay was applied to quantify the *P. gingivalis*, which infected the HGECs, in the different groups. To evaluate the expression levels of pro-inflammatory cytokines, including interleukin (IL)-1β, IL-6, and IL-8, by infected HGECs, real-time quantitative reverse transcription-polymerase chain reactions were applied. In the *in-vivo* experiments, rats were given ZA solution (ZA group) or saline (control group) by tail intravenous injection for 8 weeks. Subsequently, we put ligatures around the maxillary second molars of all the rats and inoculated *P. gingivalis* to the gingiva every other day from day 1 to day 13. The rats were sacrificed on days 3, 7, and 14 for micro-CT and histological analyses. The *in-vitro* results manifested that the quantity of *P. gingivalis* that had infected HGECs increased with the ZA concentrations. Pro-inflammatory cytokines expression by HGECs were significantly increased by 100 μM ZA. In the *in-vivo* study, compared to the control group, more *P. gingivalis* was detected in the superficial layer of gingival epithelium in the ZA group. Besides, ZA significantly increased the expression level of IL-1β on day 14 and IL-6 on days 7 and 14 in gingival tissues. These findings suggest that the oral epithelial tissues of patients who receive high-dose ZA treatment may be more susceptible to periodontal infections, resulting in severe inflammatory conditions.

## Introduction

Bisphosphonates (BPs) are a class of potent anti-resorptive agents including zoledronic acid (ZA), alendronate, risedronate, pamidronate, etidronate, and ibandronate (Lambrinoudaki et al., 2006). Due to their specific clinical efficacy, these drugs are considered first-line anti-resorptive agents and are routinely used to prevent and treat bone-related diseases such as osteoporosis, malignant tumor bone metastasis, hyperthyroidism, and Paget’s disease by systemic administration (McClung, 2003; Barbosa et al., 2021). Millions of patients received systemic administration of bisphosphonates to treat and prevent bone metabolic diseases around the world each year (Ruggiero and Drew, 2007).

Despite the effectiveness of BPs in treating bone-related diseases, long-term systematic administration of BPs could cause a rare but serious adverse effect, which is known as bisphosphonate-related osteonecrosis of the jaw (BRONJ) (Khan et al., 2015; Kishimoto et al., 2019). BRONJ is characterized by oral soft tissue healing disorders and exposure of the jaw bone, which cause necrosis of the exposed bone, severe pain, infection, fistula, or pathological jaw fracture (Sarin et al., 2008; Yarom et al., 2019). The mechanism of BRONJ has not been fully elucidated at this stage (Kishimoto et al., 2019). In addition to the inhibitory effect of BPs on bone metabolism and angiogenesis, soft tissue toxicity and oral mucosa impairment have also been identified as key factors in BRONJ (Kyrgidis and Vahtsevanos, 2009; Kumar et al., 2010). Several studies investigated that BPs have adverse effects on oral soft tissues (Scheper et al., 2009b; Kobayashi et al., 2010; Kim et al., 2011; Ravosa et al., 2011; Donetti et al., 2014; Jung et al., 2018). It has been proved that pamidronate could induce senescence of oral mucosa and impair the re-epithelialization process of oral mucosal cells (Kim et al., 2011). Long-term alendronate treatment can affect adhesion, proliferation, and terminal differentiation of healthy oral mucosa epithelial cells (Donetti et al., 2014). Besides, ZA inhibits proliferation and induces apoptosis of human gingival fibroblasts, oral epithelial cells, and keratinocytes *in-vitro* (Scheper et al., 2009a; Ravosa et al., 2011). ZA also inhibits oral epithelial cell migration, leading to delayed wound healing after tooth extraction, thereby increasing the risk of BRONJ (Kobayashi et al., 2010).

However, the previous studies merely evaluated the effects of BPs on epithelial cells under sterile conditions *in vitro*. The oral cavity has the second-largest microbiome which contains over 700 species of microbes (Paster et al., 2006). The oral mucosal epithelia contact microbes in the oral cavity directly, forming a shield for the connective tissues and bone tissues underneath, and play crucial roles in the innate immune response (Dixon et al., 2004; Tribble and Lamont, 2010; Groeger and Meyle, 2015). Periodontal pathogens infecting gingiva epithelium is a key process in the initiation and progression of periodontal diseases (Tribble and Lamont, 2010; Ji et al., 2015; Ji and Choi, 2020). Pathogenic bacteria infect gingival epithelium, then invade the underlying connective tissue, which would cause chronic destructive inflammation, and eventually lead to periodontal bone loss (Ji et al., 2015). Hence, the susceptibility of the epithelial barrier to periodontal infections deserves more attention.

To date, whether BPs affect the process of bacteria infecting the oral mucosal epithelium has not been fully elucidated. *Porphyromonas gingivalis* (*P. gingivalis*) is regarded as one of the most important pathogens associated with periodontal diseases. *P. gingivalis* can infect gingival epithelial cells, and induce inflammatory conditions in periodontal tissues (Aas et al., 2005; Ji et al., 2015; Roberts et al., 2017). In this study, the *in-vivo* and *in-vitro* experiments were designed to investigate how ZA influences the process of *P. gingivalis* infecting the oral epithelial barrier preliminarily.

## Materials and methods

### *In-vitro* model

The hTERT-immortalized cell line, human gingival epithelial cells (HGECs) (CVCL_M095), was obtained from the American Type Culture Collection (ATCC CRL-3397) and incubated in DMEM high glucose (Gibco, USA) containing 10% fetal bovine serum (Gibco, USA) at 37°C with 5% CO_2_.

*P. gingivalis* ATCC W83 (AB_2934283) used in this study was anaerobically cultured in brain heart infusion (BHI) (HuanKai, China) broth medium and blood agar plates supplemented with 5% defibrinated sheep blood (PingRui, China), 5.0 μg/ml hemin (Aladdin, China), and 1.0 μg/ml vitamin K1 (Aladdin, China). *P. gingivalis* at the mid-log phase was used in subsequent experiments.

To explore the effects of ZA on the infection process, different amounts of HGECs were seeded into culture containers of appropriate sizes depending on different experimental requirements and antibiotic-free cultured overnight. Subsequently, the bacterial suspensions of *P. gingivalis* were diluted to the corresponding concentration by using the McFarland Scale. Then HGECs were infected by *P. gingivalis* with the multiplicity of infection (MOI) of 100, treated with ZA of different concentrations (0, 1, 10, and 100 μM; MedChemExpress, USA) at the same time. The groups of the *in-vitro* study were defined as the control group (0 μM), ZA-1 group (1 μM), ZA-10 group (10 μM), and ZA-100 group (100 μM) respectively.

### Transmission electron microscope

HGECs were seeded into 6-well plates (Corning, USA) with 8 × 10^5^ cells per well and antibiotic-free cultured overnight, then infected with *P. gingivalis* at the multiplicity of infection (MOI) of 100 for 6 h. ZA of different concentrations was added as described above. Samples were gently washed 3 times with PBS to remove free *P. gingivalis*. The cells were collected using cell scrapers and fixed with 2.5% glutaraldehyde overnight at 4°C. The fixed samples were rinsed three times with 0.1M phosphate buffer (pH 7.0) for 15 min each time, followed by postfixation in 1% osmic acid solution for 1 h. After being rinsed 3 times with 0.1M phosphate buffer (pH 7.0), the samples were dehydrated with gradient concentrations of ethanol solution (including 30%, 50%, 70%, 80%, 90%, 95%, and 100%). Then the samples were treated with pure acetone for 20 min, a mixture of embedding agent and acetone (V/V = 1/1) for 1 h, a mixture of embedding agent and acetone (V/V = 3/1) for 3 h, and finally pure embedding agent overnight, after which the infiltrated samples were heated overnight at 70°C to obtain embedded samples. The samples were sliced in an ultramicrotome (LEICA EM UC7) to obtain 70 nm ultrathin sections. The sections were stained with lead citrate solution and uranyl acetate 50% ethanol saturated solution for 10 min and dried. The sections were examined using a Philips CM100 transmission electron microscope (TEM) (Philips, Amsterdam, Netherlands) to observe the condition of the *in-vitro* infection model construction.

### Immunofluorescence staining

HGECs were seeded into 15 mm coverglass bottom dishes (Biosharp, China) (2 × 10^5^ cells per dish) and antibiotic-free cultured overnight, then infected with *P. gingivalis* at the MOI of 100 for 6 h. ZA of different concentrations were added as described during the process. Wash the dishes with PBS for 2 times gently to remove the free *P. gingivalis.* After being fixed with 4% paraformaldehyde (PFA) for 10 min, *P. gingivalis* was labeled with mouse anti-*P. gingivalis* primary antibody (AB_10573813, Developmental Studies Hybridoma Bank [DSHB] hybridoma product 60BG1.3, USA 1:100 dilution), followed by Alexa Fluor 488-conjugated anti-mouse IgG secondary antibody (A0428, Beyotime, China, 1:200 dilution). HGECs were stained with Alexa Fluor 555 phalloidin (ab176756, Abcam, UK, 1:1000 dilution). The nuclei were stained with DAPI solution (Beyotime, China). Samples were analyzed with an Olympus FV 3000 confocal laser scanning microscope (Olympus, Hachioji, Japan).

To identify the internalized *P. gingivalis* and attached *P. gingivalis*, a supplementary immunofluorescence staining was performed according to the methods reported by Imamura et al. The sample preparation is performed as described above. After being fixed with 4% PFA for 10 min, extracellular *P. gingivalis* were labeled with mouse anti-*P. gingivalis* primary antibody (AB_10573813, 1:100, Developmental Studies Hybridoma Bank [DSHB] hybridoma product 60BG1.3, 3μg/ml) for 1h at room temperature. After washing 3 times gently with PBS-T, samples were incubated with DyLight 405-conjugated affinipure Goat Anti-Mouse IgG secondary antibody (H+L) (AB_2934284, EarthOx, San Francisco, CA, 1:200 dilution). After permeabilizing HGECs in 0.1% Triton X-100 solution for 10 min, internalized *P. gingivalis* were stained with the mouse anti-*P. gingivalis* primary antibody followed by Alexa Fluor 488-conjugated anti-mouse IgG secondary antibody (A0428, Beyotime, China, 1:200 dilution) (Imamura et al., 2015). HGECs were stained with Alexa Fluor 555 phalloidin (ab176756, Abcam, UK, 1:1000 dilution). Samples were observed with an LSM 980 confocal laser scanning microscope (ZEISS, Oberkochen, Germany).

### Internalization assay

After overnight antibiotic-free culture of HGECs (1 × 10^5^ cells per well) in 48-well plates (Corning, USA), the HGECs were infected with *P. gingivalis* at the MOI of 100 for 1 h, 3 h, and 6 h. ZA of different concentrations were added into the culture medium as described. After infection, the cells were washed 3 times with PBS to remove the planktonic *P. gingivalis.* To evaluate the total number of *P. gingivalis* adhering to and invading HGECs, cell lysis was performed with sterile distilled water for 30 min to release *P. gingivalis* which infected HGECs. The lysates were plated on blood agar plates by serial dilution with 7-day anaerobic incubation at 37°C until counting. For the internalized *P. gingivalis*, the cells were treated with 200 μg/mL metronidazole (Solarbio, China) and 300 μg/mL gentamicin (Solarbio, China) for 60 min to kill the extracellular bacteria before lysis. Cells were lysed in sterile water for 30 min and the lysates were plated on blood agar plates by serial dilution as described above to estimate the internalized *P. gingivalis*.

### Real-time quantitative reverse transcription-polymerase chain reaction

HGECs were seeded into in 6-well plates (Corning, USA) (8 × 10^5^ cells per well) and antibiotic-free cultured overnight, then infected with *P. gingivalis* at the MOI of 100 for 1h, 3h, and 6 h. ZA of different concentrations were added as described. Total RNA was extracted using RNA Quick Purification Kit (ES Science, China). PrimeScript RT Master Mix (TAKARA, Japan) was used to perform the reverse transcript. SYBR TB Green Premix Ex Taq II (TAKARA, Japan) was used to perform the RT-PCR assays, following the standard protocol of the manufacturer. ABI Prism 7500 Sequence Detection System was applied. Reactions were activated at 95°C for 5 min, followed by 45 PCR cycles with denaturation at 95°C for 10 s, annealing at 60°C for 10 s, and extension at 72°C for 20 s. The melting curve temperatures of each PCR gene were 95°C for 5 s and 65°C for 1 min. The relative expressions of interleukin (IL)-1β, IL-6, and IL-8 were analyzed. All quantifications were performed with β-actin as the internal standard and calculated using the 2^-ΔΔCt^ method (Livak and Schmittgen, 2001). Primer sequences used in this study were listed in **Table 1**.

**Table 1:** The sequences of RT-qPCR primer.

Gene		Sequences (5’ to 3’)
IL-1β	forward	TGAAGCAGCCATGGCAGAAG
	reverse	GGTCGGAGATTCGTAGCTGGA
IL-6	forward	AATCATCACTGGTCTTTTGGAG
	reverse	GCATTTGTGGTTGGGTCA
IL-8	forward	GACATACTCCAAACCTTTCCACC
	reverse	AACTTCTCCACAACCCTCTGC
β-actin	forward	TTGGCAATGAGCGGTT
	reverse	AGTTGAAGGTAGTTTCGTGGAT

### Animal experiments

Six-week-old female Sprague-Dawley rats (MGI:5651135) weighing 200-220 g were purchased from the Laboratory Animal Center of Sun Yat-sen University, Guangzhou, China. The rats were housed in a specified-pathogen free facility with food and water available, and fed in temperature-controlled laboratory with a 12-h light/12-h dark cycle. All procedures of this study were ethically approved by the Ethics Committee for Animal Experiments of Sun Yat-Sen University (No. SYSU-IACUC-2021-000819).

**Figure 1:**
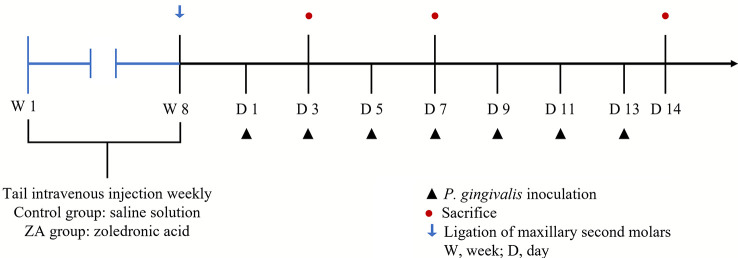
The schedule of the *in-vivo* experiment. Ligations around the cervixes of the maxillary second molars were set up on day 0 in both the control group (saline solution) and the ZA group (0.2 mg/kg ZA solution). From day 1 to day 13, 12 rats in each group received *P. gingivalis* inoculation. The blue arrow indicates the time-point of putting ligatures around the cervixes of maxillary second molars in each group; the black triangles indicate the time-points of inoculating *P. gingivalis* around the cervixes of maxillary second molars in each group; the red dots indicate the time-points of randomly euthanizing 4 rats in each group.

The procedure for the *in-vivo* experiment is shown in **Figure 1**. Rats were randomly divided into the control group (12 rats) and the ZA group (12 rats). Rats in the ZA group were given zoledronic acid (MedChemExpress, USA, 0.2 mg/kg, weekly) by tail intravenous injection for 8 weeks (Gao et al., 2021). Rats in the control group were given saline solution as blank control. After injection, ligatures of 3/0 braided silk (JinHuan, China) were placed around the cervix of the maxillary second molars of both sides of all rats while they were anesthetized with pentobarbital sodium (40 mg/kg, i.p.). After placing the ligatures, *P. gingivalis* (1 ×10^9^ CFU/mL) were inoculated in the oral cavity of all rats on days 1, 3, 5, 7, 9, 11, and 13. Four rats of each group were randomly euthanized on days 3, 7, and 14 for further experimental analysis. The isolated maxillae were fixed in 4% paraformaldehyde for 48 h for the subsequent analysis.

### Bone morphology analysis using micro-computed tomography

The isolated maxillae were scanned using micro-CT (SkyScan 1276, Bruker, Germany) under scan resolution of 15 µm, voltage of 85 kV, and current of 200 µA. The samples were aligned by using Dataviewer software (v.1.5.6.2). The bone mineral density (BMD, g/cm^3^), bone volume (BV, μm^3^), tissue volume (TV, mm^3^), and BV/TV (%) of bone around the maxillary second molar of all the samples were calculated by using CTAn software (v.1.20), and then the distances from the cemento-enamel junction to the alveolar bone crest (CEJ-ABC, μm) at the distal region of the maxillary second molars were measured.

### Hematoxylin-eosin staining

The samples were decalcified in 10% EDTA solution at 4°C for 8 weeks. Dehydration and wax leaching were performed, then the tissues were embedded in wax, sectioned at a thickness of 4 μm and mounted on glass slides. Hematoxylin and eosin (H&E) staining were used to observe the changes of periodontal soft tissues. The sections were dewaxed twice with xylene for 15 min. Gradient ethanol was used for rehydration (100% ethanol for 5 min, 75% ethanol for 5 min, 35% ethanol for 5 min). Then, the sections were stained with H&E staining kit (Servicebio, China), dehydrated (100% ethanol 3 times for 5 min each), and hyalinized with xylene twice for a total of 10 min. The sections were sealed with neutral gum. Images were captured with an Aperio slide scanner (Leica Biosystems Aperio, USA). The epithelial thickness around the second molar was measured from the 5 sites of each slice captured on Aperio ImageScope (Leika, Germany) randomly, and measured by microscopy at a magnification of 20×.

### Immunohistochemistry staining

Immunohistochemical techniques were used to detect the infection of *P. gingivalis* and the expression of inflammatory factors in the mucosal epithelial layer. The tissue sections were dewaxed and rehydrated. Then, the antigen retrieval procedure was achieved by heat induced epitope retrieval (HIER) method using 10 mM sodium citrate buffer (pH = 6.0) at 85 °C for 45 min. After antigen retrieval, all sections were treated with 3% hydrogen peroxidase for 20 min to block endogenous peroxidase activity, and 5% BSA was used for 60 minutes to seal serum. Diluted primary antibodies against *P. gingivalis* (AB_10573813, 1:100, Developmental Studies Hybridoma Bank [DSHB] hybridoma product 60BG1.3, 3μg/ml), interleukin (IL)-1β (AB_2934281, Zenbio, China, 1:100 dilution) and IL-6 (AB_2934282, Servicebio, China, 1:400 dilution) incubation were performed at 4°C overnight. Subsequently, tissue sections were incubated with anti-mouse IgG antibodies for 30 min at room temperature. The chromogenic reaction was performed by 3,3’-diaminobenzidine tetrahydrochloride (DAB, GTVisionTM + Detection System/Mo&Rb, China) chromogen substrate solution and counterstaining was performed with hematoxylin (Servicebio, China). Images were captured with an Aperio slide scanner (Leica Biosystems Aperio, USA). Region of interest (ROI) in gingival epithelium and lamina propria around the second molar was captured on Aperio ImageScope (Leika, Germany) randomly. The values of integrated option density (IOD) and the area of ROI of IL-6 and IL-1β were measured through Image-Pro Plus 7.0 (Media Cybernetics, Rockville, MD, USA). The IOD/Area values of IL-6 and IL-1β positive expression were calculated in the control group and the ZA group.

### Statistical analysis

All data are presented as the mean ± standard deviation (SD). After checking the normal distribution and the variance homogeneity of the data, the statistical differences between two groups were analyzed by an unpaired two-tailed Student’s t-test, while the statistical differences between multiple groups were evaluated by one-way ANOVA with LSD *post-hoc* test using SPSS software (version 24; SPSS Inc., IBM Corporation, Armonk, NY, USA). The significance level was set at *p* < 0.05.

## Results

### ZA promoted *P. gingivalis* infecting HGECs *in vitro* in a dose-dependent pattern

**Figure 2:**
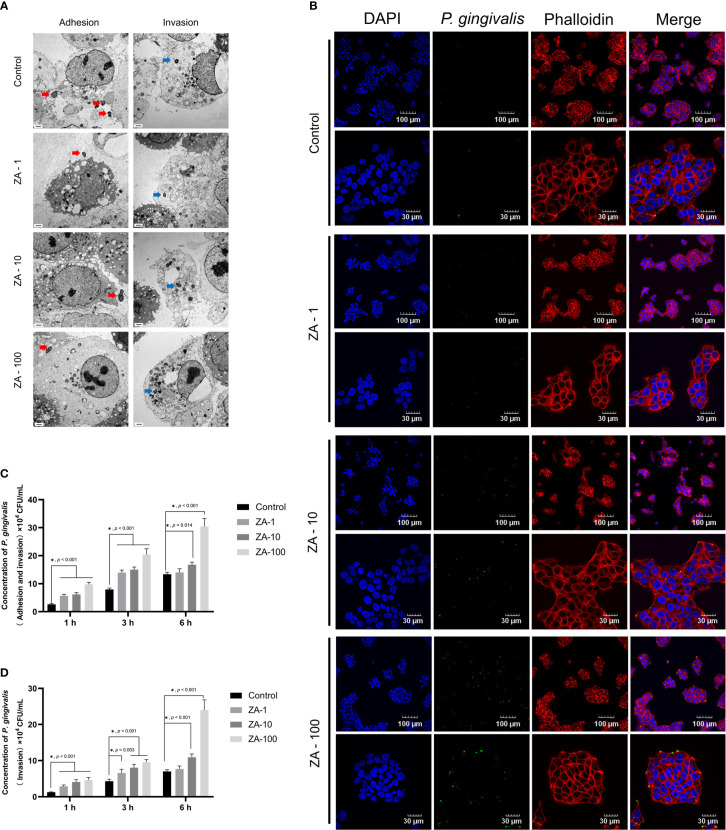
The process of *P. gingivalis* infecting HGECs in the presence of different concentrations of ZA *in vitro*. **(A)** The representative TEM images of *in-vitro* infection of HGECs by *P. gingivalis* of all groups. The red arrows indicate the *P. gingivalis* adhering to the surface of HGECs. The blue arrows indicate the *P. gingivalis* having invaded into HGECs. Scale bars = 1 μm. **(B)** The representative immunofluorescence images of *P. gingivalis* infecting HGECs for 6 h at different magnifications of all groups. The cell nucleus, blue fluorescence; the cytoskeleton, red fluorescence; *P. gingivalis*, green fluorescence. Scale bars were shown in the images. **(C)** The internalization assay results of total attached and internalized *P. gingivalis* of HGECs in all groups at 1 h, 3 h, 6 h. n = 4 at each time point. **(D)** The internalization assay results of only internalized *P. gingivalis* of HGECs in all groups at 1 h, 3 h, 6 h. n = 4 at each time point. Values represent mean ± SD. Control, ZA-1, ZA-10, ZA-100 represent 0 μM ZA, 1 μM ZA, 10 μM ZA, 100 μM ZA respectively. *p < 0.05.

TEM images showed that *P. gingivalis* not only adhered to the surface of HGECs but also invaded into the HGECs in all the groups (**Figure 2A**). We noticed that there is no significant difference among the TEM results of these groups, that may be due to the very limited field of the electron microscope.

The representative immunofluorescence images showed the *P. gingivalis* infection condition of HGECs in all groups at different magnifications (**Figures 2B**, **S1**). HGECs were clustered like paving stones. The amount of *P. gingivalis* infecting HGECs presented an increasing trend along with the increase of ZA concentration. More *P. gingivalis* were observed in the ZA-100 group than in the other groups.

The internalization assay results are shown in the **Figures 2C, D**. At 1h and 3h, the total adherent and internalized *P. gingivalis* in the ZA-1, ZA-10 and ZA-100 groups were significantly higher than that in the control group (*p* < 0.001). At 6 h, the total adherent and internalized *P. gingivalis* in the ZA-100 group was significantly higher than the other 3 groups (*p* < 0.001). After the extracellular *P. gingivalis* attached to the HGECs were killed by antibiotics treatment, the intracellular *P. gingivalis* in the 4 groups showed a similar tendency as total attached and internalized *P. gingivalis*. With the elevation of ZA concentration, the amount of internalized *P. gingivalis* and total *P. gingivalis* (both adhered and invaded) increased. The results showed that ZA has promoted the *P. gingivalis* adhesion and invasion of HGECs with a dose-dependent manner.

### High concentration of ZA increased the expression levels of pro-inflammatory factors by HGECs infected by *P. gingivalis*

**Figure 3:**
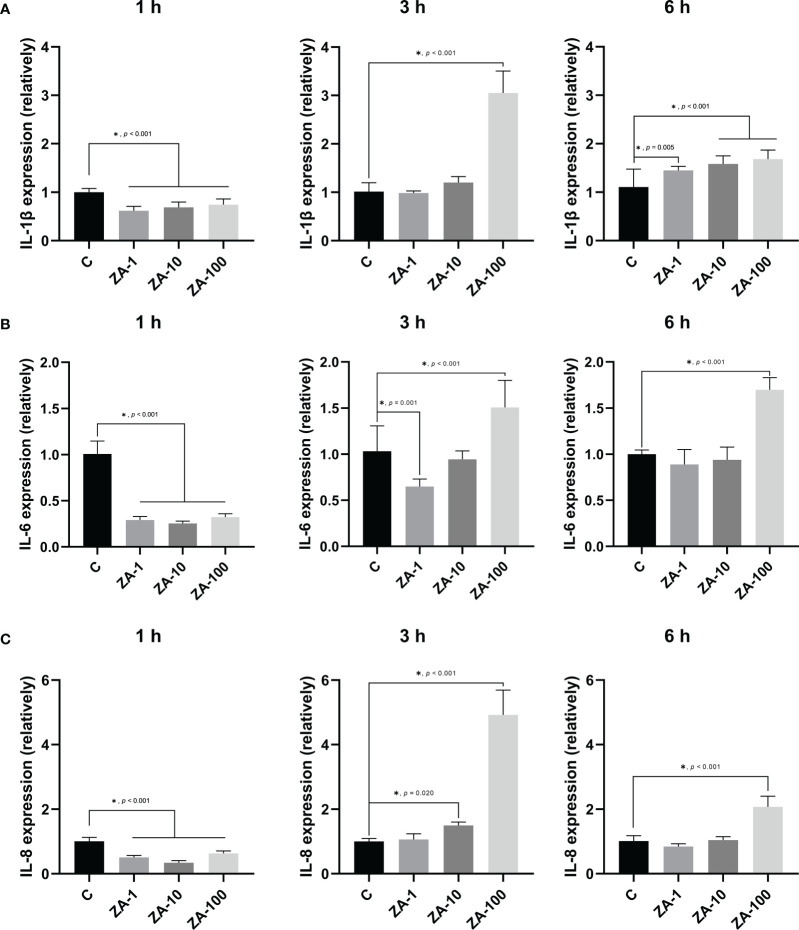
The relative mRNA expression levels of pro-inflammatory factors of HGECs. The relative mRNA expression levels of IL-1β **(A)**, IL-6 **(B)**, and IL-8 **(C)** of HGECs after *P. gingivalis* infection for 1 h, 3 h, and 6 h of the 4 groups. n = 4 in each group. Values represent mean ± SD. Control, ZA-1, ZA-10, ZA-100 represent 0 μM ZA, 1 μM ZA, 10 μM ZA, 100 μM ZA respectively. *p < 0.05.

The expression levels of pro-inflammatory factors of HGECs were analyzed by RT-qPCR. At 1 h, the relative expression levels of IL-1β by HGECs in the experimental groups were significantly lower than that in the control group, which means that ZA inhibited the expression of IL-1β by HGECs at the early stage of infection. At 3 h, the expressional levels of IL-1β in the ZA-1 and ZA-10 groups were not significantly different from that in the control group, while the expression of IL-1β in the ZA-100 group was significantly higher than that in the other groups (*p* < 0.001). At 6 h, the expression levels of IL-1β in the ZA-1, ZA-10 and ZA-100 groups were significantly higher than that in the control group, and the expression levels of IL-1β elevated with the increase of ZA concentration (**Figure 3A**).

Similarly, ZA inhibited the expression of IL-6 by HGECs at 1 h. The expression levels of IL-6 in the ZA-1, ZA-10 and ZA-100 groups were significantly lower than that in the control group at 1 h. At 3 h, the expression level of IL-6 in the ZA-1 group significantly decreased. At 6 h, the expression levels of IL-6 in both the ZA-1 and ZA-10 groups were not significantly different from that in the control group. However, at 3 h and 6 h, the expression levels of IL-6 in the ZA-100 group were significantly higher than that in the control group (**Figure 3B**).

At 1 h, similar to IL-1β, the expression levels of IL-8 in the ZA-1, ZA-10 and ZA-100 groups were significantly lower than that in the control group, which indicated that ZA inhibited the expression of IL-8. At 3 h, the expression levels of IL-8 in the ZA-10 and ZA-100 groups were significantly higher than that in the control group, but the expression level of IL-8 in the ZA-1 group was not significantly different from that in the control group. At 6 h, the expression level of IL-8 in the ZA-100 group was significantly higher than that in the control group, while the expression levels of IL-8 in the ZA-1 and ZA-10 groups were not significantly different from that in the control group (**Figure 3C**).

### ZA significantly increased the alveolar bone mineral density in rats

**Figure 4:**
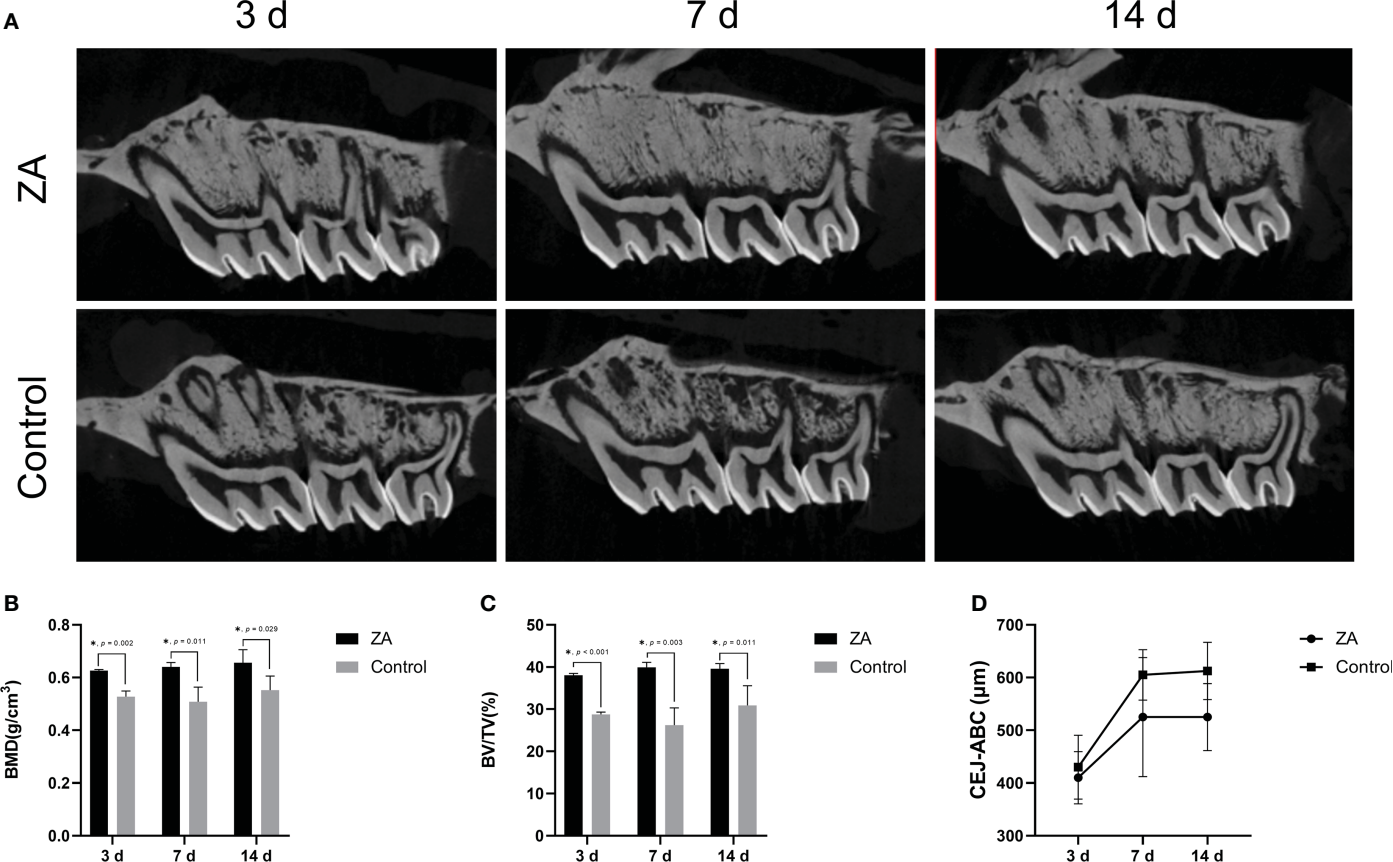
Micro-CT analysis showed bone morphology parameters of the ZA group and the control group. **(A)** Representative micro-CT sagittal images of maxillary alveolar bone surrounding the first and second molars on days 3, 7, and 14. The ZA group showed higher bone density and less alveolar bone resorption than the control group. ***(*B*)*** The bone mineral density (BMD) of maxillary alveolar bone surrounding the second molars on days 3, 7, and 14. n = 4 at each time point. **(C)** The bone volume per total volume (BV/TV) of maxillary alveolar bone surrounding the second molars on days 3, 7, and 14. n = 4 at each time point. **(D)** The distances between the cemento-enamel junction and alveolar bone crest (CEJ-ABC) of maxillary alveolar bone surrounding the second molars on days 3, 7, and 14. n = 4 at each time point. Values represent mean ± SD. *p < 0.05.

The intraoral images of the ligations around maxillary second molars are shown in **Figure S2**. Representative micro-CT sagittal images of maxillary alveolar bones are presented in **Figure 4A**. The average bone mineral density (BMD) and the bone volume per total volume (BV/TV) of maxillary alveolar bone surrounding the maxillary second molars in the ZA group were significantly higher than that in the control group on days 3,7, and 14 (**Figures 4B, C**). These results indicated that the intravenous injection of ZA significantly increased the alveolar bone mineral density of rats, and the animal models were successfully established. We evaluated the cemento-enamel junction and alveolar bone crest (CEJ-ABC) distances of the 2 groups (**Figure 4D**). On days 3, 7, and 14, the mean CEJ-ABC distance of the ZA group tended to be larger than that of the control group, although there was no significant difference between the two groups. The alveolar bone level tended to be stable from day 7 to day 14 in both groups during the entire experiment.

### ZA promoted *P. gingivalis* infecting the gingival epithelium of rats

**Figure 5:**
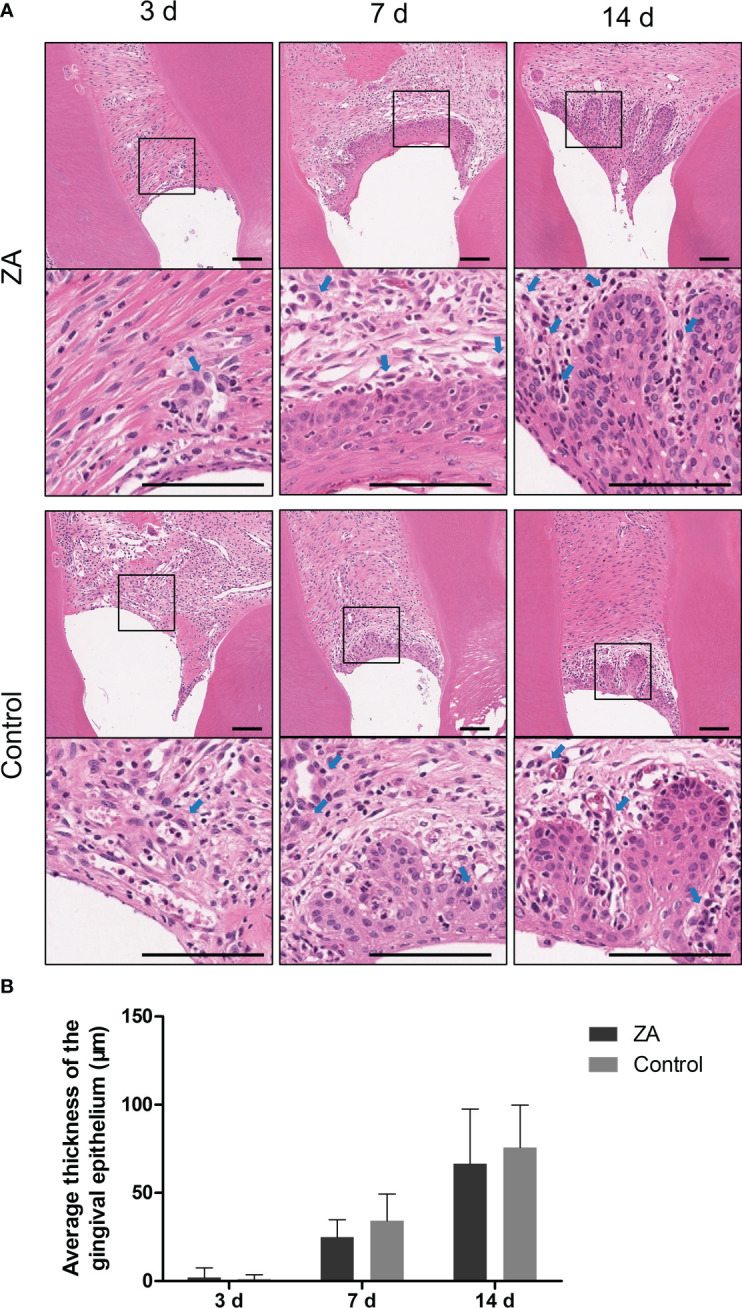
Result of histology staining of the ZA group and the control group. **(A)** Representative histology staining images of the ZA group and the control group. The images in the second panel of each group are enlarged views of the boxed areas in the above images. Blue arrows indicated the leukocytes. Scale bars = 100 μm. **(B)** Mean thickness of gingival epithelium in the ZA group and the control group on days 3, 7, and 14. n = 4 at each time point. Values represent mean ± SD.

The representative images of hematoxylin and eosin (H&E) staining are shown in **Figure 5**. From day 3 to day 14, the gingival epithelia became thicker. The average thicknesses of epithelia are shown in **Figure 5B**. On day 7, short epithelial rete pegs appeared in some of the gingival epithelia. On day 14, the epithelial rete pegs extended obviously. The epithelial thicknesses between the control group and the ZA group showed no significant difference on day 7 and day 14. The inflammatory cell infiltration in the epithelia and proper lamina of both groups can be detected on days 3,7 and 14.

**Figure 6:**
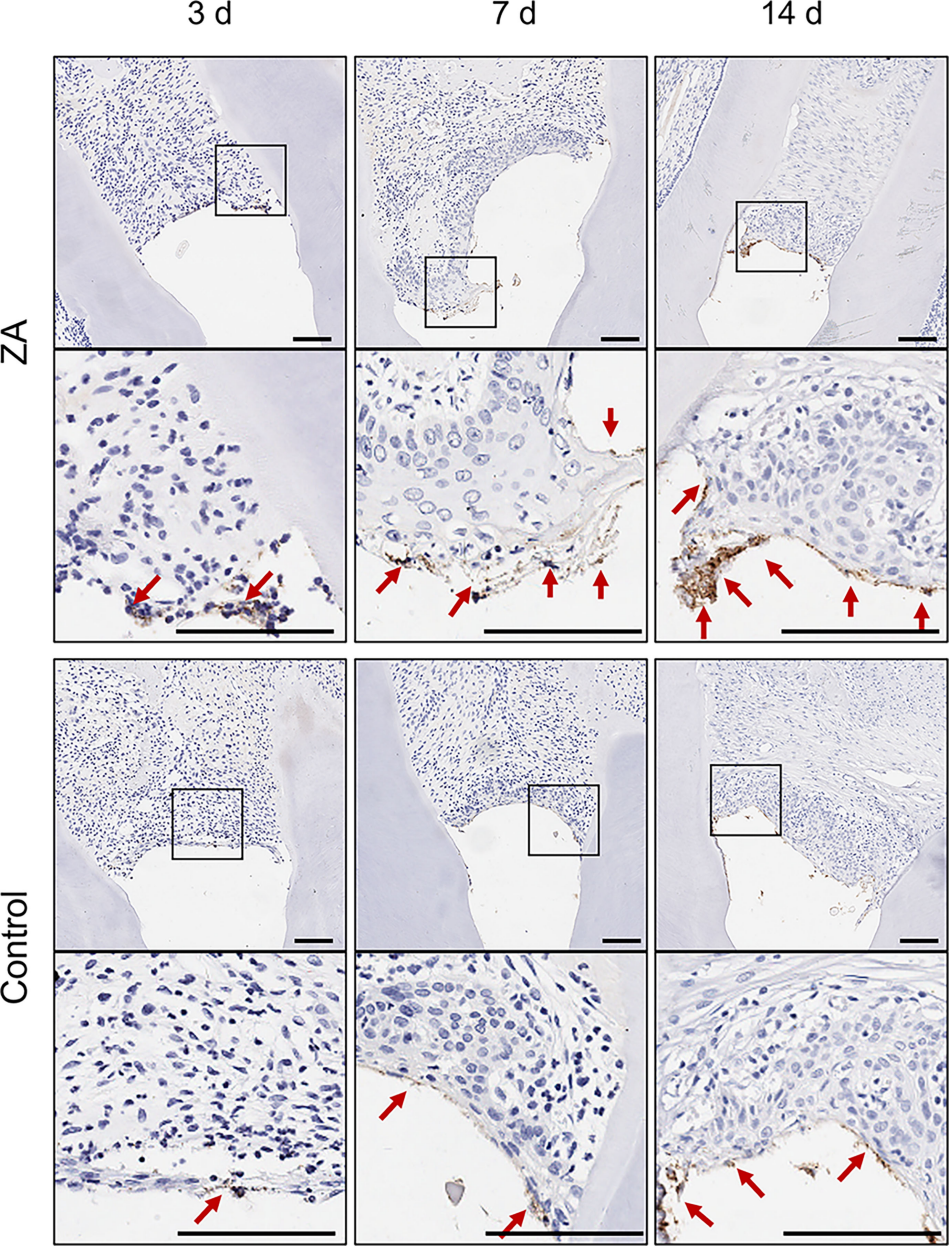
*P. gingivalis* in gingival tissues of the ZA group and the control group. The images in the second panel of each group are enlarged views of the boxed areas in the above images. Red arrows indicated the *P. gingivalis* infect the gingival epithelial layer. Scale bars = 100 μm.

*P. gingivalis* infections of gingival tissues were observed by *P. gingivalis*-stained sections of the ZA group and the control group on days 3, 7, and 14 (**Figure 6**). *P. gingivalis* were detected in the superficial gingival epithelial layer in both groups throughout the *in-vivo* experiment. Few bacteria were found in the lamina propria. On days 3, 7, and 14, more adherent *P. gingivalis* were found in the ZA group than in the control group.

### ZA changed the levels of pro-inflammatory factors in the gingival tissues infected by *P. gingivalis*

**Figure 7:**
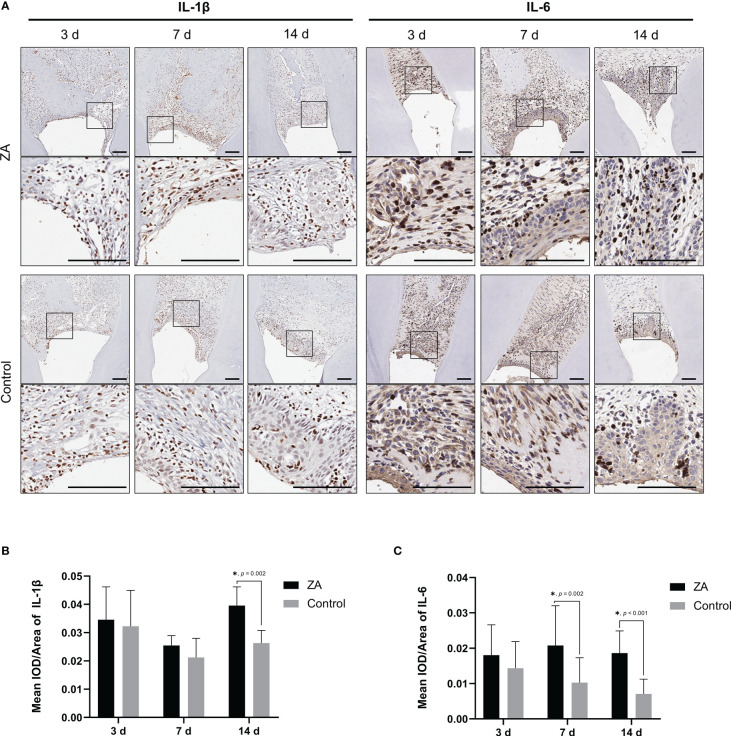
Expression of IL-1β and IL-6 in the ZA group and the control group. **(A)** Representative immunohistochemical staining of IL-1β and IL-6 in gingival tissue sections of both groups. The images in the second panel of each group are enlarged views of the boxed areas in the above images. Scale bars = 100 μm. **(B)** Mean IOD/Area of IL-1β in the ZA group and the control group on days 3, 7, and 14. n = 4 at each time point. **(C)** Mean IOD/Area of IL-6 in the ZA group and the control group on days 3, 7, and 14. n = 4 at each time point. Values represent mean ± SD. *p < 0.05.

To evaluate the inflammation level of gingival tissues in the 2 groups, immunohistochemical staining (**Figure 7A**) and semi-quantitative evaluation (**Figures 7B, C**) were applied to analyze the expression of IL-1β and IL-6 on days 3, 7 and 14. On day 14, the expression level of IL-1β in the ZA group was significantly higher than that in the control group (*p* = 0.002). And the expression levels of IL-6 in the ZA group were significantly higher than that of the control group on days 7 (*p* = 0.002) and 14 (*p* < 0.001).

## Discussion

Long-term BPs treatment could bring additional risks to the oral epithelium. According to our *in-vitro* experiment results, ZA could facilitate *P. gingivalis* infecting the gingival epithelium at the early stage, and this effect presented a ZA dose-dependent manner.

​At the same time point, the results of the internalization assay showed an increasing tendency for the amount of attached and internalized *P. gingivalis* in HGECs, which increases with ZA concentration. The quantities of *P. gingivalis* that had adhered to and invaded HGECs in the ZA-10 and ZA-100 group were significantly higher than the control group at all the time points we detected. It can be concluded that with the same MOI of infection, higher concentrations of ZA caused more serious *P. gingivalis* infection in the gingival epithelial cells. The *in-vivo* studies showed a similar trend that more *P. gingivalis* were observed in the ZA group than in the control group. It can be inferred that ZA could decrease the anti-infection ability of the gingival epithelial cells. To date, there is no proper method to quantitatively assess the *P. gingivalis* infection *in vivo*, which may need further exploration.

Previous studies have investigated the effect of ZA on non-infected oral epithelial cells. Although the concentrations of ZA and treatment times were varied among these studies, the conclusions were generally consistent. ZA would decrease the cell viability of oral epithelial cells (Scheper et al., 2009a; Ravosa et al., 2011; Ohnuki et al., 2012; Pabst et al., 2012; Arai et al., 2013; Pabst et al., 2015; Wang et al., 2019). In addition, ZA could inhibit the proliferation of oral epithelial cells (Scheper et al., 2009a; Ohnuki et al., 2012; McLeod et al., 2014) and induce apoptosis of oral epithelial cells (Scheper et al., 2009b; Pabst et al., 2012; Pabst et al., 2015; Wang et al., 2019). An *in-vivo* study also showed that ZA promoted the expression of caspase 3, a marker of apoptosis, in oral epithelial cells (Allam et al., 2011). ZA had similar effects on gingival fibroblasts including inhibiting proliferation (McLeod et al., 2014) and inducing apoptosis (Agis et al., 2010; Ravosa et al., 2011; Jung et al., 2018). It can be concluded that BPs were toxic and harmful to oral soft tissues. This may be some of the possible reasons why ZA promoted *P. gingivalis* to infect gingival epithelial cells.

It has been reported that ZA could reduce the oral epithelial thickness (Bullock et al., 2020). In an *in-vitro* 3D-tissue engineering model of oral mucosa, 1μM and 10 μM ZA could reduce epithelial thickness in a dose-dependent manner. Although there was no difference in the thickness of epithelial layer between the control group and the experimental group on days 3, 7, and 14, in our results, there existed a trend that the average epithelial thickness of ZA group were thinner than the control group. The reasons for this result may be due to the differences between the *in-vitro* 3D-tissue engineering models and *in-vivo* environment. The concentration of ZA and *P. gingivalis* may also influence the epithelial thickness. The specific reasons still need further studies.

In addition to promoting *P. gingivalis* infection of gingival epithelial cells, ZA may also affect the expression of pro-inflammatory cytokines by epithelial cells which were stimulated by *P. gingivalis*. *P. gingivalis*, as a typical periodontal pathogen, could induce infected epithelial cells and immune cells to produce diverse pro-inflammatory cytokines and chemokines, such as IL-1β, IL-6, IL-8 (Groeger and Meyle, 2019). The pro-inflammatory cytokines recruit inflammatory cells and initiate the subsequent inflammatory response, leading to periodontal soft tissue destruction at the early phase of periodontitis (Cekici et al., 2014).

Notably, in the *in-vitro* experiments, the expression levels of IL-1β, IL-6, and IL-8 of infected gingival epithelial cells in all the experimental groups decreased at 1 h. That is to say, at the initial stage of infection, ZA significantly inhibited the expression of pro-inflammatory cytokines by the infected epithelial cells. Yet, at 3 h and 6 h, significantly increased expression levels of IL-1β, IL-6, and IL-8 were detected in the ZA-100 group. An *in-vivo* study proved that long-term ZA treatment would elevate the expression levels of pro-inflammatory cytokines (TNF-α and IL-1β) in the gingiva of rats (de Barros Silva et al., 2017). However, in our study, after long-term ZA administration, the *in-vivo* results showed no significant difference in IL-1β levels between the control and experimental groups on days 3 and 7, while the experimental group had significantly higher expression levels of IL-1β at day 14 than the control group. There was no difference in the expression levels of IL-6 between the control group and the experimental group on day 3 while the expression level of IL-6 in the experimental group was significantly higher than that in the control groups on days 7 and 14. The current results suggest that periodontal epithelial cells in patients with long-term, high-dose ZA application are more likely to produce pro-inflammatory factors when stimulated by periodontal pathogens, and these patients may develop inflammatory state in periodontal soft tissues more easily than others.

Periodontal diseases are inflammatory conditions caused by subgingival and supragingival microbial plaque in the soft and hard tissues surrounding teeth. The main manifestations of periodontal diseases are periodontal soft tissue inflammation, probing bleeding, alveolar bone resorption and loss of attachment (Kinane, 2001; Kinane et al., 2017). BPs has a strong anti-resorption effect so that BPs not only are used to treat diseases related to bone metabolism (Barbosa et al., 2021), but also had a therapeutic potential in periodontal diseases (Kc et al., 2021; Zymperdikas et al., 2021; Arena et al., 2022). Application of BPs in periodontal disease can inhibit osteoclast recruitment and induce osteoclasts apoptosis, thereby reducing alveolar bone resorption caused by periodontal tissue inflammation. Several clinical investigations have demonstrated that BPs could be benefit to the clinical periodontal condition (Pradeep et al., 2012; Sharma and Pradeep, 2012a; Sharma and Pradeep, 2012b; Pradeep et al., 2013; Bhavsar et al., 2016; Kanoriya et al., 2016; Taguchi et al., 2019). Researchers proved that systemic application of BPs significantly benefited the periodontal pocket depth (PD) and clinical attachment level (CAL) in postmenopausal women with chronic periodontitis who simultaneously received mechanical periodontal treatments (Bhavsar et al., 2016). ZA can significantly prevent symptomatic periodontal diseases and reduce teeth loss in patients with osteoporosis, on the condition that they have maintained good oral hygiene (Taguchi et al., 2019). In addition, local administration is thought to be more acceptable than systemic administration because it causes fewer adverse effects (Needleman et al., 1995; Position Paper: The Role of Controlled Drug Delivery for Periodontitis, 2000; Zymperdikas et al., 2021). Compared to placebo, 1% alendronate gel for local delivery could significantly improve CAL and reduce PD after treating periodontal diseases by scaling and root planning (SRP) (Pradeep et al., 2012; Sharma and Pradeep, 2012a; Sharma and Pradeep, 2012b; Pradeep et al., 2013).

Although using BPs in treating periodontal diseases are beneficial to the clinical periodontal outcomes, few studies have focused on its effect on gingival soft tissues. Consistent with previous studies (Pradeep et al., 2012; Sharma and Pradeep, 2012a; Sharma and Pradeep, 2012b; Pradeep et al., 2013; Bhavsar et al., 2016; Taguchi et al., 2019), we observed that systemic administration of ZA increased the BMD and BV/TV significantly, and there was a tendency to reduce alveolar bone resorption. Nevertheless, we also found that in the rats of the ZA group, more *P. gingivalis* were observed in the gingival epithelia, and higher levels of multiple pro-inflammatory cytokines were detected. It can be speculated that ZA could increase the risk of infection in periodontal soft tissues and promote the inflammation level while the dental plaque is not effectively controlled. Therefore, the periodontal mechanical treatment is of vital importance. Maintaining patients’ oral hygiene and reducing the intraoral bacteria could be one of the indispensable preconditions for using BPs to treat periodontal diseases.

The concentration of BPs may be one of the key factors in the treatment of periodontal disease. According to the current results, ZA promote *P. gingivalis* infecting gingival epithelial cells in a dose-dependent manner. Moreover, high concentration of ZA significantly promoted the expression of pro-inflammatory factors in the gingival epithelium while low concentration of ZA did not. Previous studies have shown that high-dose of ZA could impair cell viability, induce apoptosis and reduce the osteogenic differentiation of periodontal ligament stem cells (Di Vito et al., 2020). It can be concluded that high-dose of ZA could be harmful to periodontal tissues. Further studies are needed to explore the safe and effective concentrations of BPs for the treatment of periodontal diseases. Our results also suggested that oral hygiene instruction and regular dental examination should be conducted for patients who are receiving ZA treatment for systemic diseases, especially malignant tumor bone metastasis. For the patients receiving high-dose ZA treatment and simultaneously suffering from periodontal infection, enhanced measures should be taken to control bacterial infection, for instance, considering the strategy of antibiotic delivery.

In summary, ZA weakened the resistance of the periodontal epithelial barrier to *P. gingivalis*. In addition, high-dose ZA treatment increased the expression of proinflammatory cytokines by gingival epithelial cells stimulated by *P. gingivali*s. High-dose ZA treatment may increase the susceptibility of periodontal epithelial barrier to periodontal pathogens infection, which causes an inflammatory state in periodontal soft tissues. Hence, it is necessary for the patients who have long-term and high-doses ZA treatment to maintain good oral hygiene, so as to keep periodontal soft tissues health.

## Conclusion

In conclusion, we have established and characterized *in-vitro* and *in-vivo* models of epithelial barrier infection by *P. gingivalis*, and evaluated the role played by ZA in the early stages of epithelial barrier infection. We have confirmed that ZA promoted *P. gingivalis* infecting epithelial cells in a dose-dependent manner, and high concentrations of ZA may increase proinflammatory cytokines expression by epithelial cells. These findings suggested that the periodontal epithelial tissues of patients receiving high-dose ZA treatment may be more susceptible to periodontal diseases, and the expression levels of pro-inflammatory cytokines in periodontal soft tissues of these patients would elevate, which would develop inflammatory states in periodontal tissues.

## Data availability statement

The original contributions presented in the study are included in the article/**Supplementary Materials**. Further inquiries can be directed to the corresponding authors.

## Ethics statement

The animal study was reviewed and approved by Ethics Committee for Animal Experiments of Sun Yat-Sen University.

## Author contributions

HS, PL and QK performed the *in-vitro* and *in-vivo* experiments. HS and PL analyzed the data. XY and FD conceived and designed this study and interpreted the results. HS, PL, QK, FD and XY wrote the manuscript. All authors contributed to the article and approved the submitted version.

## References

[B1] Aas J. A. Paster B. J. Stokes L. N. Olsen I. Dewhirst F. E. (2005). Defining the normal bacterial flora of the oral cavity. J. Clin. Microbiol. 43 (11), 5721–5732. doi: 10.1128/jcm.43.11.5721-5732.2005 16272510 PMC1287824

[B2] Agis H. Blei J. Watzek G. Gruber R. (2010). Is zoledronate toxic to human periodontal fibroblasts? J. Dent. Res. 89 (1), 40–45. doi: 10.1177/0022034509354298 19948943

[B3] Arai N. Inoue S. Tomihara K. Tsuno H. Noguchi M. (2013). *In vitro* synergistic effects of zoledronic acid and calcium on viability of human epithelial cells. Oral. Dis. 19 (2), 200–205. doi: 10.1111/j.1601-0825.2012.01971.x 22891943

[B4] Allam E. Allen M. Chu T, M. Ghoneima H. Jack Windsor L. (2011). *In vivo* effects of zoledronic acid on oral mucosal epithelial cells. Oral. Dis. 17 (3), 291–7. doi: 10.1111/j.1601-0825.2010.01739.x 20860766 PMC3010441

[B5] Arena C. Caponio V. C. A. Zhurakivska K. Lo Russo L. Lo Muzio L. Troiano G. (2022). Added effect of 1% topical alendronate in intra-bony and inter-radicular defects as part of step II periodontal therapy: a systematic review with meta-analysis and trial sequential analysis. BMC Oral. Health 22 (1), 15. doi: 10.1186/s12903-022-02044-1 35062940 PMC8780760

[B6] (2000). Position paper: The role of controlled drug delivery for periodontitis. J. Periodontol 71 (1), 125–140. doi: 10.1902/jop.2000.71.1.125 29537535

[B7] Barbosa J. S. Almeida Paz F. A. Braga S. S. (2021). Bisphosphonates, old friends of bones and new trends in clinics. J. Med. Chem. 64 (3), 1260–1282. doi: 10.1021/acs.jmedchem.0c01292 33522236

[B8] Bhavsar N. V. Trivedi S. R. Dulani K. Brahmbhatt N. Shah S. Chaudhri D. (2016). Clinical and radiographic evaluation of effect of risedronate 5 mg as an adjunct to treatment of chronic periodontitis in postmenopausal women (12-month study). Osteoporos Int. 27 (8), 2611–2619. doi: 10.1007/s00198-016-3577-8 27026334

[B9] Bullock G. Miller C. McKechnie A. Hearnden V. (2020). Synthetic hydroxyapatite inhibits bisphosphonate toxicity to the oral mucosa *In vitro* . Materials (Basel) 13 (9), 2086. doi: 10.3390/ma13092086 32369961 PMC7254283

[B10] Cekici A. Kantarci A. Hasturk H. Van Dyke T. E. (2014). Inflammatory and immune pathways in the pathogenesis of periodontal disease. Periodontol 2000 64 (1), 57–80. doi: 10.1111/prd.12002 24320956 PMC4500791

[B11] de Barros Silva P. G. Ferreira Junior A. E. C. de Oliveira C. C. Brizeno L. A. C. Wong D. V. T. Lima Junior R. C. P.. (2017). Chronic treatment with zoledronic acid increases inflammatory markers in periodontium of rats. J. Oral. Pathol. Med. 46 (10), 1046–1053. doi: 10.1111/jop.12640 28865081

[B12] Di Vito A. Chiarella E. Baudi F. Scardamaglia P. Antonelli A. Giudice D.. (2020). Dose-dependent effects of zoledronic acid on human periodontal ligament stem cells: An *In vitro* pilot study. Cell Transplant. 29, 963689720948497. doi: 10.1177/0963689720948497 33086890 PMC7784504

[B13] Dixon D. R. Bainbridge B. W. Darveau R. P. (2004). Modulation of the innate immune response within the periodontium. Periodontol 2000 35, 53–74. doi: 10.1111/j.0906-6713.2004.003556.x 15107058

[B14] Donetti E. Gualerzi A. Sardella A. Lodi G. Carrassi A. Sforza C. (2014). Alendronate impairs epithelial adhesion, differentiation and proliferation in human oral mucosa. Oral. Dis. 20 (5), 466–472. doi: 10.1111/odi.12154 23837876

[B15] Gao S. Y. Lin R. B. Huang S. H. Liang Y. J. Li X. Zhang S. E.. (2021). PDGF-BB exhibited therapeutic effects on rat model of bisphosphonate-related osteonecrosis of the jaw by enhancing angiogenesis and osteogenesis. Bone 144, 115117. doi: 10.1016/j.bone.2019.115117 31676407

[B16] Groeger S. E. Meyle J. (2015). Epithelial barrier and oral bacterial infection. Periodontol 2000 69 (1), 46–67. doi: 10.1111/prd.12094 26252401

[B17] Groeger S. Meyle J. (2019). Oral mucosal epithelial cells. Front. Immunol. 10. doi: 10.3389/fimmu.2019.00208 PMC6383680 30837987

[B18] Imamura K. Kokubu E. Kita D. Ota K. Ishihara K. Saito A. (2015). Cigarette smoke condensate modulates migration of human gingival epithelial cells and their interactions with porphyromonas gingivalis. J. Periodontal Res. 50 (3), 411–421. doi: 10.1111/jre.12222 25196284

[B19] Ji S. Choi Y. (2020). Microbial and host factors that affect bacterial invasion of the gingiva. J. Dent. Res. 99 (9), 1013–1020. doi: 10.1177/0022034520922134 32392459

[B20] Ji S. Choi Y. S. Choi Y. (2015). Bacterial invasion and persistence: critical events in the pathogenesis of periodontitis? J. Periodontal Res. 50 (5), 570–585. doi: 10.1111/jre.12248 25487426

[B21] Jung J. Park J. S. Righesso L. Pabst A. M. Al-Nawas B. Kwon Y. D.. (2018). Effects of an oral bisphosphonate and three intravenous bisphosphonates on several cell types *in vitro* . Clin. Oral. Investig. 22 (7), 2527–2534. doi: 10.1007/s00784-018-2349-6 29388023

[B22] Kanoriya D. Pradeep A. R. Singhal S. Garg V. Guruprasad C. N. (2016). Synergistic approach using platelet-rich fibrin and 1% alendronate for intrabony defect treatment in chronic periodontitis: A randomized clinical trial. J. Periodontol 87 (12), 1427–1435. doi: 10.1902/jop.2016.150698 27562221

[B23] Kc K. Bhattarai B. P. Shrestha S. Shrestha B. Shrestha M. (2021). EFFECT OF LOCALLY DELIVERED BISPHOSPHONATES ON ALVEOLAR BONE: A SYSTEMATIC REVIEW AND META-ANALYSIS. J. Evid Based Dent. Pract. 21 (3), 101580. doi: 10.1016/j.jebdp.2021.101580 34479678

[B24] Khan A. A. Morrison A. Hanley D. A. Felsenberg D. McCauley L. K. O’Ryan F.. (2015). Diagnosis and management of osteonecrosis of the jaw: a systematic review and international consensus. J. Bone Miner Res. 30 (1), 3–23. doi: 10.1002/jbmr.2405 25414052

[B25] Kim R. H. Lee R. S. Williams D. Bae S. Woo J. Lieberman M.. (2011). Bisphosphonates induce senescence in normal human oral keratinocytes. J. Dent. Res. 90 (6), 810–816. doi: 10.1177/0022034511402995 21427353 PMC3144120

[B26] Kinane D. F. (2001). Causation and pathogenesis of periodontal disease. Periodontol 2000 25, 8–20. doi: 10.1034/j.1600-0757.2001.22250102.x 11155179

[B27] Kinane D. F. Stathopoulou P. G. Papapanou P. N. (2017). Periodontal diseases. Nat. Rev. Dis. Primers 3, 17038. doi: 10.1038/nrdp.2017.38 28805207

[B28] Kishimoto H. Noguchi K. Takaoka K. (2019). Novel insight into the management of bisphosphonate-related osteonecrosis of the jaw (BRONJ). Jpn Dent. Sci. Rev. 55 (1), 95–102. doi: 10.1016/j.jdsr.2018.09.002 31193410 PMC6526304

[B29] Kobayashi Y. Hiraga T. Ueda A. Wang L. Matsumoto-Nakano M. Hata K.. (2010). Zoledronic acid delays wound healing of the tooth extraction socket, inhibits oral epithelial cell migration, and promotes proliferation and adhesion to hydroxyapatite of oral bacteria, without causing osteonecrosis of the jaw, in mice. J. Bone Miner Metab. 28 (2), 165–175. doi: 10.1007/s00774-009-0128-9 19882100

[B30] Kumar S. K. Gorur A. Schaudinn C. Shuler C. F. Costerton J. W. Sedghizadeh P. P. (2010). The role of microbial biofilms in osteonecrosis of the jaw associated with bisphosphonate therapy. Curr. Osteoporos Rep. 8 (1), 40–48. doi: 10.1007/s11914-010-0008-1 20425090

[B31] Kyrgidis A. Vahtsevanos K. (2009). Increased risk for bisphosphonate-related osteonecrosis of the jaws in patients wearing dentures could be attributable to impaired mucosal cell wound healing. J. Oral. Maxillofac. Surg. 67 (6), 1355–1356. doi: 10.1016/j.joms.2008.05.368 19446234

[B32] Lambrinoudaki I. Christodoulakos G. Botsis D. (2006). Bisphosphonates. Ann. N Y Acad. Sci. 1092, 397–402. doi: 10.1196/annals.1365.036 17308164

[B33] Livak K. J. Schmittgen T. D. (2001). Analysis of relative gene expression data using real-time quantitative PCR and the 2(-delta delta C(T)) method. Methods 25 (4), 402–408. doi: 10.1006/meth.2001.1262 11846609

[B34] McClung M. R. (2003). Bisphosphonates. Endocrinol. Metab. Clin. North Am. 32 (1), 253–271. doi: 10.1016/s0889-8529(02)00079-8 12699302

[B35] McLeod N. M. Moutasim K. A. Brennan P. A. Thomas G. Jenei V. (2014). *In vitro* effect of bisphosphonates on oral keratinocytes and fibroblasts. J. Oral. Maxillofac. Surg. 72 (3), 503–509. doi: 10.1016/j.joms.2013.08.007 24342576

[B36] Needleman I. G. Pandya N. V. Smith S. R. Foyle D. M. (1995). The role of antibiotics in the treatment of periodontitis (Part 2–controlled drug delivery). Eur. J. Prosthodont Restor. Dent. 3 (3), 111–117. 8603153

[B37] Ohnuki H. Izumi K. Terada M. Saito T. Kato H. Suzuki A.. (2012). Zoledronic acid induces s-phase arrest *via* a DNA damage response in normal human oral keratinocytes. Arch. Oral. Biol. 57 (7), 906–917. doi: 10.1016/j.archoralbio.2011.11.015 22172403

[B38] Pabst A. M. Krüger M. Ziebart T. Jacobs C. Sagheb K. Walter C. (2015). The influence of geranylgeraniol on human oral keratinocytes after bisphosphonate treatment: An *in vitro* study. J. Craniomaxillofac Surg. 43 (5), 688–695. doi: 10.1016/j.jcms.2015.03.014 25913629

[B39] Pabst A. M. Ziebart T. Koch F. P. Taylor K. Y. Al-Nawas B. Walter C. (2012). The influence of bisphosphonates on viability, migration, and apoptosis of human oral keratinocytes–*in vitro* study. Clin. Oral. Investig. 16 (1), 87–93. doi: 10.1007/s00784-010-0507-6 21225298

[B40] Paster B. J. Olsen I. Aas J. A. Dewhirst F. E. (2006). The breadth of bacterial diversity in the human periodontal pocket and other oral sites. Periodontol 2000 42, 80–87. doi: 10.1111/j.1600-0757.2006.00174.x 16930307

[B41] Pradeep A. R. Kumari M. Rao N. S. Naik S. B. (2013). 1% alendronate gel as local drug delivery in the treatment of class II furcation defects: a randomized controlled clinical trial. J. Periodontol 84 (3), 307–315. doi: 10.1902/jop.2012.110729 22554293

[B42] Pradeep A. R. Sharma A. Rao N. S. Bajaj P. Naik S. B. Kumari M. (2012). Local drug delivery of alendronate gel for the treatment of patients with chronic periodontitis with diabetes mellitus: a double-masked controlled clinical trial. J. Periodontol 83 (10), 1322–1328. doi: 10.1902/jop.2012.110292 22264208

[B43] Ravosa M. J. Ning J. Liu Y. Stack M. S. (2011). Bisphosphonate effects on the behaviour of oral epithelial cells and oral fibroblasts. Arch. Oral. Biol. 56 (5), 491–498. doi: 10.1016/j.archoralbio.2010.11.003 21146154

[B44] Roberts J. S. Atanasova K. R. Lee J. Diamond G. Deguzman J. Hee Choi C.. (2017). Opportunistic pathogen porphyromonas gingivalis modulates danger signal ATP-mediated antibacterial NOX2 pathways in primary epithelial cells. Front. Cell Infect. Microbiol. 7. doi: 10.3389/fcimb.2017.00291 PMC5495830 28725637

[B45] Ruggiero S. L. Drew S. J. (2007). Osteonecrosis of the jaws and bisphosphonate therapy. J. Dent. Res. 86 (11), 1013–1021. doi: 10.1177/154405910708601101 17959890

[B46] Sarin J. DeRossi S. S. Akintoye S. O. (2008). Updates on bisphosphonates and potential pathobiology of bisphosphonate-induced jaw osteonecrosis. Oral. Dis. 14 (3), 277–285. doi: 10.1111/j.1601-0825.2007.01381.x 18336375

[B47] Scheper M. A. Badros A. Chaisuparat R. Cullen K. J. Meiller T. F. (2009a). Effect of zoledronic acid on oral fibroblasts and epithelial cells: a potential mechanism of bisphosphonate-associated osteonecrosis. Br. J. Haematol 144 (5), 667–676. doi: 10.1111/j.1365-2141.2008.07504.x 19036117 PMC2739302

[B48] Scheper M. A. Badros A. Salama A. R. Warburton G. Cullen K. J. Weikel D. S.. (2009b). A novel bioassay model to determine clinically significant bisphosphonate levels. Support Care Cancer 17 (12), 1553–1557. doi: 10.1007/s00520-009-0710-7 19653010

[B49] Sharma A. Pradeep A. R. (2012a). Clinical efficacy of 1% alendronate gel as a local drug delivery system in the treatment of chronic periodontitis: a randomized, controlled clinical trial. J. Periodontol 83 (1), 11–18. doi: 10.1902/jop.2011.110091 21542734

[B50] Sharma A. Pradeep A. R. (2012b). Clinical efficacy of 1% alendronate gel in adjunct to mechanotherapy in the treatment of aggressive periodontitis: a randomized controlled clinical trial. J. Periodontol 83 (1), 19–26. doi: 10.1902/jop.2011.110206 21609254

[B51] Taguchi A. Shiraki M. Tanaka S. Ohshige H. Nakamura T. (2019). Improved periodontal disease and prevention of tooth loss in osteoporosis patients receiving once-yearly zoledronic acid: a randomized clinical trial. Menopause 26 (11), 1277–1283. doi: 10.1097/gme.0000000000001393 31688575

[B52] Tribble G. D. Lamont R. J. (2010). Bacterial invasion of epithelial cells and spreading in periodontal tissue. Periodontol 2000 52 (1), 68–83. doi: 10.1111/j.1600-0757.2009.00323.x 20017796 PMC3647226

[B53] Wang Q. Liu J. Guo T. Liu D. Pan J. (2019). Epidermal growth factor reverses the inhibitory effects of the bisphosphonate, zoledronic acid, on human oral keratinocytes and human vascular endothelial cells *In vitro via* the epidermal growth factor receptor (EGFR)/Akt/Phosphoinositide 3-kinase (PI3K) signaling pathway. Med. Sci. Monit 25, 700–710. doi: 10.12659/msm.911579 30675875 PMC6357820

[B54] Yarom N. Shapiro C. L. Peterson D. E. Van Poznak C. H. Bohlke K. Ruggiero S. L.. (2019). Medication-related osteonecrosis of the jaw: MASCC/ISOO/ASCO clinical practice guideline. J. Clin. Oncol. 37 (25), 2270–2290. doi: 10.1200/jco.19.01186 31329513

[B55] Zymperdikas V. F. Yavropoulou M. P. Kaklamanos E. G. Papadopoulos M. A. (2021). Bisphosphonates as supplement to dental treatment: A network meta-analysis. J. Dent. Res. 100 (4), 341–351. doi: 10.1177/0022034520972945 33208008

