# Supplementary material for: Zoledronic acid affects the process of Porphyromonas gingivalis infecting oral mucosal epithelial barrier: An in-vivo and in-vitro study
Source: Front Cell Infect Microbiol. 2023 Mar 28;13:1104826. doi: 10.3389/fcimb.2023.1104826 (PMC10086244; doi:10.3389/fcimb.2023.1104826)
Supplement: Supplementary file 1 [file DataSheet_1.docx]

**FIGURE S1** The representative immunofluorescence images of *P. gingivalis* attaching to and having invaded in HGECs at different magnifications of all groups. The attached *P. gingivalis* were stained blue and the internalized *P. gingivalis* were stained green. The cytoskeletons of HGECs were stained red. The right images of each group are enlarged views of the boxed areas in the left images. The Scale bars = 25 μm.


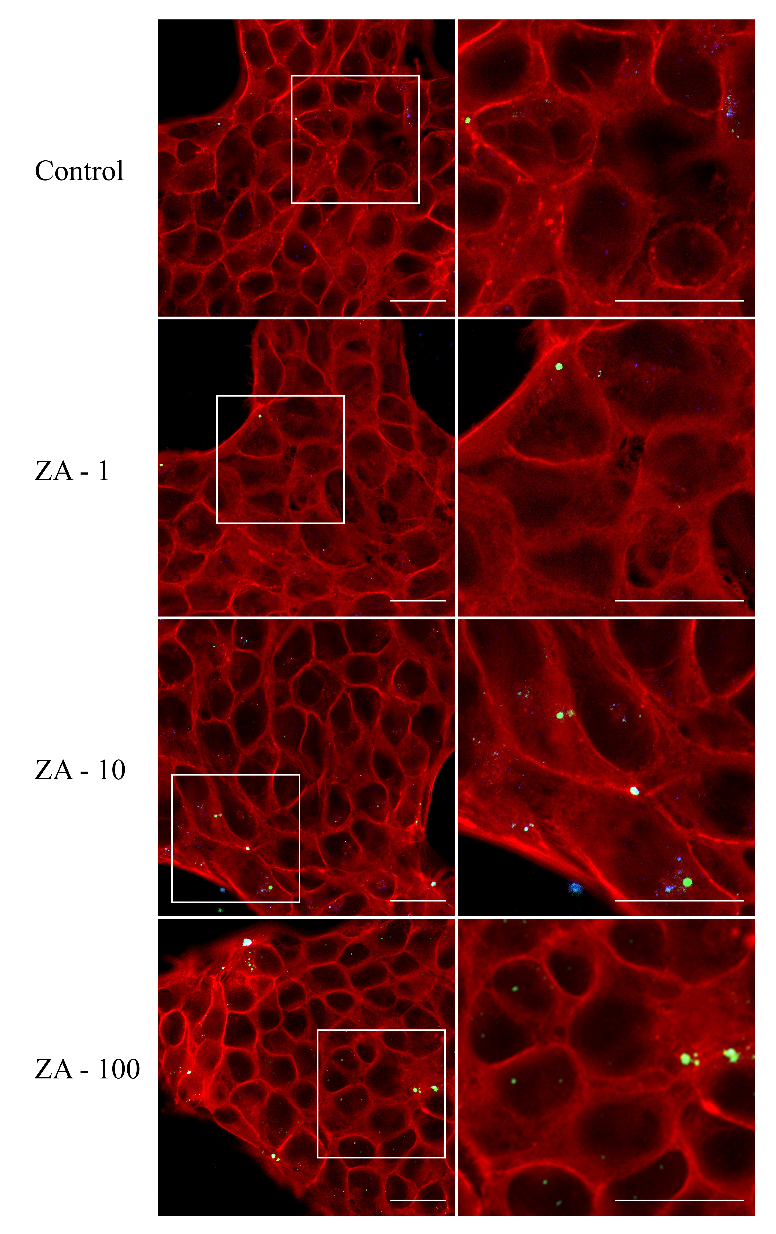


**FIGURE S2** The intraoral images of the ligations around maxillary second molars.

**
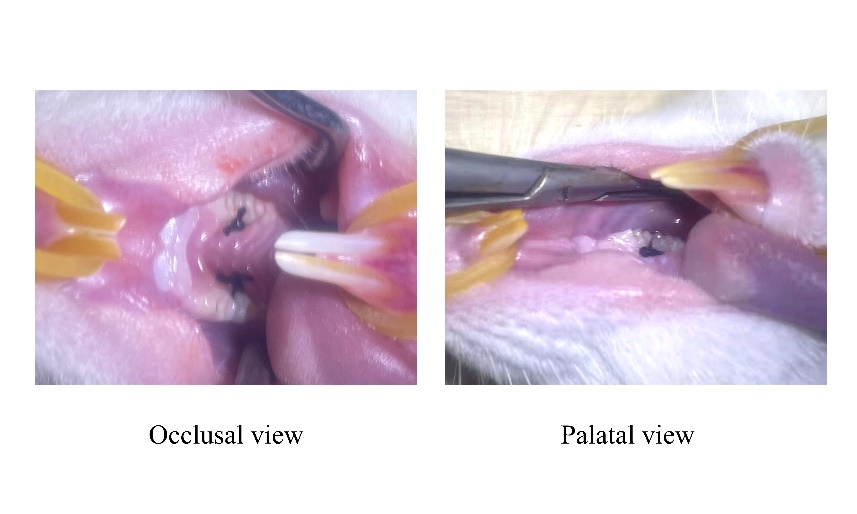
**
